# Supplementary material for: Biological Activities and Chemical Composition of Santolina africana Jord. et Fourr. Aerial Part Essential Oil from Algeria: Occurrence of Polyacetylene Derivatives
Source: Molecules. 2019 Jan 8;24(1):204. doi: 10.3390/molecules24010204 (PMC6337488; doi:10.3390/molecules24010204)
Supplement: Supplementary file 1 [file molecules-24-00204-s001.pdf]

Table 1. Chemical composition of 18 samples of *Santolina africana* aerial part essential oil.

N°	Components	RI [a]	B1	B2	B3	B4	B5	B6	F1	F2	F3	F4	F5	F6	H1	H2	H3	H4	H5	H6
1	Hexanal	774	tr	-	tr	-	tr	tr	-	-	tr	-	-	-	tr	tr	tr	tr	tr	tr
2	Santolina triene	901	-	tr	-	-	0.1	0.5	0.5	0.5	0.8	0.3	0.1	-	0.2	0.1	tr	0.2	0.4	0.5
3	α-Thujene	923	tr	-	tr	-	tr	0.1	-	tr	tr	-	-	-	tr	0.1	tr	0.1	tr	tr
4	α-Pinene	931	1.0	1.0	1.1	1.6	0.5	1.0	0.8	0.9	1.2	0.4	1.2	0.6	0.9	1.4	1.0	1.3	1.1	0.6
5	Camphene	944	-	-	0.2	0.6	0.3	0.2	0.5	0.2	0.7	0.5	0.6	0.2	0.3	0.7	0.2	0.2	0.3	0.2
6	1-Octen-3-ol	961	tr	-	-	-	tr	tr	-	-	tr	-	-	-	tr	tr	-	tr	tr	tr
7	Sabinene	965	0.7	0.5	0.7	0.3	0.6	1.1	0.5	0.7	0.7	0.4	0.8	0.2	1.0	1.3	0.8	1.2	0.7	1.0
8	β-Pinene	971	14.0	18.7	14.8	2.4	2.5	6.5	12.7	12.7	17.0	6.3	17.6	5.5	6.1	8.5	11.4	7.2	4.4	5.4
9	Myrcene	981	9.4	8.7	11.3	4.2	11.0	14.0	10.8	13.6	18.9	8.2	9.2	5.6	11.3	12.5	20.9	7.6	5.4	7.3
10	Yomogi alcohol	985	-	0.1	-	-	tr	tr	0.1	0.1	0.1	0.1	-	-	tr	tr	-	tr	0.1	0.1
11	α-Terpinene	1009	0.1	-	-	-	0.3	0.3	0.0	-	0.1	-	-	-	0.1	0.1	0.2	0.1	-	0.1
12	*p*-Cymene	1012	0.7	0.6	1.1	0.8	0.6	0.7	0.7	1.3	1.1	0.7	1.0	0.5	1.3	1.6	1.4	1.0	0.9	0.7
13	1,8-Cineole*	1021	10.1	6.7	13.7	11.4	8.6	12.9	9.5	10.1	9.1	8.9	9.5	5.0	13.6	12.7	14.4	16.8	14.5	16.6
14	Limonene*	1021	0.1	0.1	0.2	0.0	0.1	0.1	0.1	0.2	0.1	0.1	0.2	-	0.2	0.2	0.2	0.3	0.3	0.2
15	β-Phellandrene*	1021	0.3	0.3	0.3	tr	0.2	0.2	0.3	0.3	0.4	0.3	0.3	0.2	0.4	0.3	0.4	0.2	0.2	0.2
16	Santolina alcohol*	1021	tr	14.0	0.8	0.5	0.2	1.2	3.4	4.5	2.6	2.0	0.9	2.4	4.1	0.2	1.6	4.0	4.2	11.5
17	γ-Terpinene	1048	0.2	-	-	-	0.6	0.4	0.1	-	0.1	-	tr	-	0.2	0.2	0.3	0.4	0.1	0.5
18	*trans*-Sabinene hydrate	1054	0.1	0.1	0.1	0.3	0.1	0.2	0.2	0.1	0.2	0.2	0.4	0.2	0.2	0.2	0.1	0.5	0.5	0.3
19	Artemisia alcohol	1069	0.1	0.3	tr	-	tr	0.1	0.2	0.1	0.1	tr	-	-	0.1	tr	0.1	0.1	0.1	0.2
20	Terpinolene	1078	tr	-	tr	0.1	0.1	0.1	-	tr	tr	-	-	-	tr	0.1	0.1	0.1	tr	0.1
21	Linalool	1084	0.3	0.3	0.3	0.2	tr	0.4	0.3	0.0	0.3	0.3	0.4	0.8	0.4	0.4	0.4	0.7	0.4	0.4
22	2-Methylbutyl 2-methylbutyrate	1086	tr	0.2	0.2	0.1	tr	0.1	0.1	0.2	0.1	0.1	0.1	-	0.1	0.1	0.1	0.1	0.1	0.1
23	Lyratal	1099	-	2.7	0.1	-	0.2	1.0	0.9	0.8	0.7	1.9	0.2	-	1.1	0.3	tr	0.2	0.9	0.5
24	Chrysanthenone	1101	0.1	0.5	-	-	tr	1.1	0.1	0.3	0.1	tr	tr	4.5	0.5	0.5	0.7	0.1	0.1	tr
25	α-Campholenal	1105	0.1	-	-	0.2	tr	tr	tr	-	tr	-	tr	0.1	tr	0.1	tr	0.1	tr	tr
26	Limonaketone	1106	0.2	-	0.2	-	-	-	-	tr	-	-	-	-	-	-	-	0.1	-	0.1
27	*p*-*cis*-Menth-2-ene-1-ol	1108	-	tr	0.1	-	0.1	0.1	0.1	-	0.1	-	0.1	0.1	0.1	0.1	0.1	0.2	0.1	0.1
28	Camphor	1121	tr	0.2	0.9	7.9	0.9	0.7	4.5	0.7	3.5	3.5	4.2	1.8	1.7	2.3	1.1	0.9	1.8	1.3
29	*trans*-Pinocarveol	1124	0.3	0.6	0.6	1.8	0.2	0.2	0.3	0.3	0.2	0.2	0.4	0.4	0.4	0.2	0.3	0.4	0.5	0.3
30	Verbenol	1129	0.3	0.2	0.1	-	0.1	0.2	0.1	0.1	0.1	0.1	0.1	0.5	0.3	0.2	0.3	0.4	0.4	0.1
31	Pinocarvone	1135	tr	-	tr	-	tr	tr	-	tr	-	-	tr	-	0.1	0.1	tr	tr	tr	0.1
32	Lyratol	1140	0.3	1.3	0.2	0.1	1.1	2.1	4.6	2.1	3.3	6.7	1.1	0.9	2.2	1.1	0.1	1.3	4.4	1.9
33	Ethyl benzoate	1144	0.4	0.3	0.2	0.1	0.2	0.2	0.1	-	0.1	0.1	-	-	0.2	0.2	0.2	-	0.1	tr

34	*cis*-Chrysanthenol	1147	16.5	8.4	1.3	0.7	4.7	3.2	3.8	4.6	2.7	6.0	5.2	3.9	8.3	10.6	7.4	9.9	12.7	2.2
35	Borneol	1149	-	-	-	0.4	0.9	0.2	0.9	0.3	0.7	0.5	0.4	0.2	0.4	0.6	0.1	0.4	1.2	0.3
36	*p*-Cymen-8-ol	1158	0.1	-	tr	-	tr	tr	-	-	-	-	-	-	0.1	0.1	0.1	0.1	0.1	0.1
37	Terpinen-4-ol	1162	7.3	2.0	1.7	3.1	1.8	2.1	2.8	1.6	2.2	1.4	1.8	2.9	3.8	2.9	4.5	3.9	2.6	2.1
38	Myrtenal	1170	0.2	0.4	0.4	0.5	0.1	0.1	0.2	0.2	0.1	0.1	0.2	0.1	0.2	0.2	0.2	0.2	0.2	0.2
39	α-Terpineol	1172	1.3	0.6	0.5	0.5	0.4	0.6	0.7	0.4	0.5	0.4	0.6	0.5	0.8	0.5	0.7	0.7	0.7	0.6
40	Myrtenol	1179	0.2	0.3	0.3	0.4	0.1	0.1	0.2	0.1	0.1	0.1	0.2	0.0	0.3	0.2	0.2	0.3	0.4	0.2
41	Verbenone	1182	0.2	0.1	-	-	-	tr	-	-	-	tr	-	tr	0.1	tr	0.1	0.1	0.0	
42	Cuminaldehyde	1212	0.1	0.1	0.1	0.1	tr	tr		0.1		0.1	tr	-	0.1	0.1	0.1	0.1	0.1	0.1
43	Chrysanthenyl acetate	1245	0.3	0.2	0.1	-	0.1	tr		0.1	tr	0.1	-	0.1	0.1	0.2	0.1	0.1	0.2	tr
44	Lyratyl acetate	1253	-	0.2	-	-	0.1	0.2	0.5	0.2	0.3	1.2	0.1	-	0.2	0.1	-	0.1	0.2	0.1
45	Bornyl acetate	1263	0.1	-	0.1	0.1	-	tr	0.1	0.1	0.1	0.1	0.1	-	0.2	0.1	0.1	0.1	0.1	0.1
46	Lavandulyl acetate	1265	-	-	-	-	-	-	0.1	tr	tr	0.1	-	-	0.1	0.0	-	-	0.1	tr
47	Carvacrol	1276	0.2	0.1	0.1	0.2	0.1	tr	0.1	0.1	0.1	0.1	0.1	0.2	0.1	0.1	0.1	0.1	0.1	tr
48	Eugenol	1327	0.2	-	-	-	0.1	0.1	-	-	-	-	-	-	0.1	0.1	0.1	0.1	0.1	0.2
49	(*Z*)-Jasmone	1367	0.1	0.1	0.1	0.2	0.1	tr	0.1	0.2	0.1	0.1	0.1	0.1	0.2	0.1	0.2	0.2	0.2	0.1
50	Methyl eugenol	1370	0.1		0.1	0.1	tr	-	-	0.1	tr	tr	0.0	0.2	0.1	0.1	0.0	0.1	0.2	0.1
51	α-Copaene	1375	0.1	tr	0.1	-	0.4	0.4	0.1	0.1	0.1	0.2	0.1	0.1	0.1	0.1	0.1	0.1	0.1	0.1
52	β-Elemene	1387	0.2	0.2	0.1	-	0.5	0.8	0.2	0.3	0.2	0.4	0.2	0.5	0.2	0.1	0.2	tr	tr	0.1
53	(*E*)-β-Caryophyllene	1416	0.1	0.1	tr	-	1.0	0.8	0.2	0.2	0.2	0.2	0.2	1.0	0.2	0.2	0.2	0.4	0.1	0.1
54	β-Copaene	1432	-	-	0.1	0.3	0.4	0.4	0.1	0.2	0.1	0.1	0.2	0.1	0.1	0.1	0.1	0.1	0.1	0.1
55	*trans*-α-Bergamotene	1435	-	-	-	-	tr	tr	-	-	-	-	-	-	-	-	-	-	-	-
56	(*E*)-β-Farnesene	1447	0.1	-	tr	-	0.4	0.2	tr	0.1	tr	0.1	0.1	0.2	0.1	0.1	0.1	0.1	tr	tr
57	α-Humulene	1456	0.1	-	0.1	-	0.2	0.1	tr	0.1	0.1	tr	-	-	-	-	-	-	-	-
58	Capillene	1458	0.1	-	0.4	0.1	0.4	tr	0.1	0.1	0.1	0.2	tr	-	3.7	4.1	0.2	6.6	7.5	16.9
59	α-Curcumene	1469	0.6	0.4	0.5	1.4	1.8	1.0	1.4	1.3	1.1	1.4	1.1	3.2	0.5	0.7	0.4	0.6	0.3	0.4
60	γ-Curcumene	1472	0.3	-	-	-	2.6	1.1	0.1	-	0.1	-	-	0.3	0.1	0.1	0.1	0.1	-	0.1
61	Germacrene D	1475	3.5	0.1	0.1	-	25.3	20.2	5.0	1.5	4.2	3.8	2.0	7.5	2.1	2.0	3.5	1.9	1.2	2.7
62	Myristicine	1486	0.6	0.3	0.3	0.5	0.1	0.1	0.3	0.2	0.2	0.3	0.3	0.6	0.2	0.1	0.1	0.1	0.2	0.1
63	Bicyclogermacrene	1490	0.7	0.1	0.1	-	6.3	2.5	0.7	0.1	0.6	0.4	0.2	0.3	0.2	0.2	0.5	0.3	0.1	0.4
64	δ-Cadinene	1513	0.2	tr	0.1	-	0.3	0.3	0.2	0.1	0.2	0.2	0.1	0.4	0.1	0.2	0.1	0.1	0.1	0.1
65	Elemicine	1521	0.1	-	-	-	tr	tr	0.1	0.1	0.1	0.1	0.1	0.4	-	0.1	tr	-	-	tr
66	β-Elemol*	1533	1.9	1.0	tr	-	0.7	1.3	2.0	0.7	1.4	0.1	2.0	3.5	1.6	1.3	1.4	0.5	1.1	0.9
67	(*E*)-α-Bisabolene*	1533	0.6	-	0.3	0.3	4.5	4.3	0.7	1.0	0.6	0.1	1.4	1.0	1.1	0.9	0.7	1.1	0.6	0.8
68	Salviadienol	1539	0.1	0.2	0.3	0.4	0.1	0.1	0.2	0.3	0.2	0.3	0.2	0.7	0.2	0.2	0.1	0.2	0.2	0.1
69	Hexenyl benzoate	1543	0.2	0.4	0.2	0.4	0.0	0.1	0.1	tr	tr	0.1	0.1	0.2	0.1	0.1	0.1	-	tr	tr
70	β-Calacorene	1548	tr	0.1	0.1	0.2	tr	tr	0.1	tr	0.1	0.1	0.1	0.1	tr	0.0	tr	0.1	0.1	tr

71	Spathulenol	1563	7.4	9.1	15.1	20.7	4.3	2.5	8.7	9.4	6.4	11.6	8.0	9.9	6.2	6.4	5.3	5.2	6.0	3.5
72	1,5-Epoxy-salvial-4(14)-ene	1568	0.2	-	0.7	-	0.4	0.3	-	-	-	0.3	-	-	-	-	-	-	-	-
73	Caryophyllene oxide	1569	0.2	0.6	0.8	1.2	0.1	0.1	0.5	0.9	0.3	0.5	0.5	1.6	0.3	0.4	0.3	1.1	0.4	0.2
74	Salvia-4(14)-en-1-one	1577	0.1	0.3	0.3	0.5	0.1	0.1	0.2	0.3	0.2	0.3	0.3	0.6	0.2	0.2	0.1	0.2	0.2	0.1
75	Dill apiol	1589	0.6	0.1	0.4	0.4	tr	tr	0.3	-	0.3	0.1	0.1	0.7	0.3	-	0.2	0.2	0.1	tr
76	Capillin	1596	-	-	-	-	-	-	tr	0.2	-	-	0.5	-	0.6	0.7	-	0.5	1.2	1.2
77	γ-Eudesmol	1617	0.4	0.8	0.2	0.1	0.2	0.2	0.3	0.7	0.3	0.3	0.6	0.9	0.4	0.3	0.3	0.2	0.4	0.2
78	iso-Spathulenol	1620	0.5	0.3	0.4	0.3	0.3	0.2	0.6	0.5	0.4	0.6	0.4	1.4	0.4	0.4	0.3	0.4	0.4	0.3
79	τ-Muurolol	1624	0.4	0.5	0.6	0.8	0.0	0.2	0.7	0.6	0.5	0.8	0.4	1.4	0.4	0.5	0.4	0.0	0.5	0.3
80	δ-Cadinol	1628	0.1	-	0.3	-	-	-	0.1	tr	0.1	0.1	-	-	0.4	0.1	0.1	0.5	0.1	0.3
81	β-Eudesmol	1633	2.4	3.0	-	tr	1.1	0.5	0.7	0.7	0.5	1.4	1.2	1.8	0.9	0.6	2.1	0.8	0.9	0.3
82	α-Cadinol	1637	0.2	0.2	0.3	0.4	0.1	0.1	0.2	0.4	0.1	0.5	0.1	0.4	tr	0.1	0.3	0.2	0.3	tr
83	α-Eudesmol	1638	0.2	-	-	-	0.0	0.1	-	-	tr	-	tr	0.3	0.1	tr	0.1	-	0.2	tr
84	α-Bisabolol oxide	1640	tr	0.1	0.7	1.7	0.0	-	-	0.1	tr	0.1	tr	-	tr	0.2	0.2	0.5	0.5	0.3
85	β-Bisabolol	1660	0.1	-	-	-	tr	tr	tr	0.1	tr	0.1	0.1	0.1	0.1	0.1	0.1	tr	0.1	0.1
86	α-Bisabolol	1666	3.6	2.6	13.2	20.0	4.5	2.2	4.7	9.1	3.5	8.3	6.9	6.6	6.6	7.6	4.4	6.8	7.4	7.8
87	Eudesma-4-(15)-7-dien-1-β-ol	1670	0.2	-	tr	-	tr	-	tr	0.2	tr	tr	tr	0.4	tr	tr	tr	-	-	-
88	(*E*)- 2-(2',4'-hexadiynylidene)-1,6-dioxaspiro[4.4]-nona-3,7-diene	1757	2.2	3.8	1.1	3.8	1.2	0.7	3.0	4.7	2.2	4.3	7.3	4.5	1.8	2.3	1.6	0.9	2.0	0.7
89	(*Z*)-2-(2',4'-hexadiynylidene)-1,6-dioxaspiro[4.4]-nona-3,7-diene	1796	0.2	tr	-	-	0.1	0.0	0.1	0.2	0.1	0.2	0.4	0.2	0.1	0.1	0.0	-	0.1	tr
90	(*Z*)-Tonghaosu	1823	0.2	-	0.1	-	tr	tr	0.1	0.1	0.1	0.2	0.1	0.1	0.1	0.2	0.1	tr	0.0	tr
91	(*E*)-Tonghaosu	1828	0.5	0.2	3.6	1.5	0.4	0.6	3.1	2.2	2.3	3.8	1.1	3.7	2.1	2.6	1.5	0.6	1.2	0.3
	Total (%)		94.6	94.4	92.7	94.1	95.1	93.6	95.4	94.7	95.7	93.0	93.2	92.4	93.3	94.6	95.2	92.5	94.9	95.2
	Yield		0.06	0.03	0.06	0.03	0.15	0.17	0.05	0.08	0.12	0.05	0.07	0.03	0.09	0.08	0.14	0.10	0.13	0.07

Order of elution and percentages of components are given on apolar column, except those with an asterisk*: percentage on polar column. RI [a]: retention indices on apolar column. Stations of collection of *S. africana*: F: Fesdis. B: Bouilef. H: Hamla. tr: Trace.

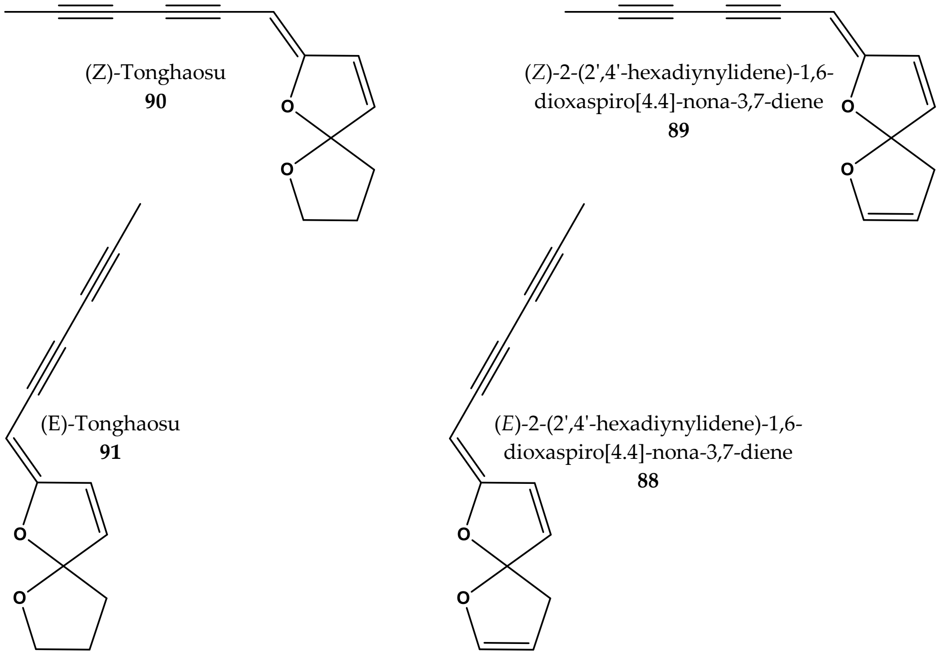

Figure 1. Structure of polyacetylenes derivatives 88, 89, 90 and 91.

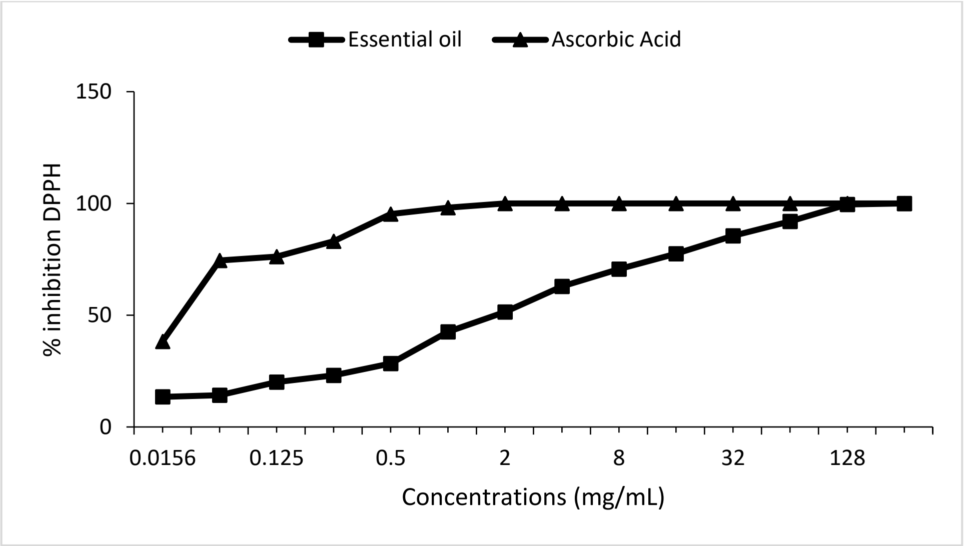

Figure 2. Antioxidant test of *Santolina africana* essential oil and ascorbic acid against DPPH•.
